# Specialized Intercellular Communications via Tunnelling Nanotubes in Acute and Chronic Leukemia

**DOI:** 10.3390/cancers14030659

**Published:** 2022-01-28

**Authors:** Alessandro Allegra, Mario Di Gioacchino, Gabriella Cancemi, Marco Casciaro, Claudia Petrarca, Caterina Musolino, Sebastiano Gangemi

**Affiliations:** 1 Department of Human Pathology in Adulthood and Childhood “Gaetano Barresi”, Division of Hematology, University of Messina, 98125 Messina, Italy; gabcan17@gmail.com (G.C.); cmusolino@unime.it (C.M.); 2 Center for Advanced Studies and Technology, G.d’Annunzio University, 66100 Chieti, Italy; claudia.petrarca@unich.it; 3 Institute for Clinical Immunotherapy and Advanced Biological Treatments, 65100 Pescara, Italy; 4 Allergy and Clinical Immunology Unit, Department of Clinical and Experimental Medicine, University of Messina, 98125 Messina, Italy; marco.casciaro@unime.it (M.C.); gangemis@unime.it (S.G.); 5 Department of Medicine and Aging Sciences, G.d’Annunzio University, 66100 Chieti, Italy

**Keywords:** tunneling nanotubes, cell communication, cancer, hematologic malignancies, leukemia, multiple myeloma, chemoresistance, miRNAs, mitochondrial transfer

## Abstract

**Simple Summary:**

Tunneling nanotubes (TNTs) are cytoplasmic channels which regulate the contacts between cells and allow the transfer of several elements, including ions, mitochondria, microvesicles, exosomes, lysosomes, proteins, and microRNAs. Through this transport, TNTs are implicated in different physiological and pathological phenomena, such as immune response, cell proliferation and differentiation, embryogenesis, programmed cell death, and angiogenesis. TNTs can promote cancer progression, transferring substances capable of altering apoptotic dynamics, modifying the metabolism and energy balance, inducing changes in immunosurveillance, or affecting the response to chemotherapy. In this review, we evaluated their influence on hematologic malignancies’ progression and resistance to therapies, focusing on acute and chronic myeloid and acute lymphoid leukemia.

**Abstract:**

Effectual cell-to-cell communication is essential to the development and differentiation of organisms, the preservation of tissue tasks, and the synchronization of their different physiological actions, but also to the proliferation and metastasis of tumor cells. Tunneling nanotubes (TNTs) are membrane-enclosed tubular connections between cells that carry a multiplicity of cellular loads, such as exosomes, non-coding RNAs, mitochondria, and proteins, and they have been identified as the main participants in healthy and tumoral cell communication. TNTs have been described in numerous tumors in in vitro, ex vivo, and in vivo models favoring the onset and progression of tumors. Tumor cells utilize TNT-like membranous channels to transfer information between themselves or with the tumoral milieu. As a result, tumor cells attain novel capabilities, such as the increased capacity of metastasis, metabolic plasticity, angiogenic aptitude, and chemoresistance, promoting tumor severity. Here, we review the morphological and operational characteristics of TNTs and their influence on hematologic malignancies’ progression and resistance to therapies, focusing on acute and chronic myeloid and acute lymphoid leukemia. Finally, we examine the prospects and challenges for TNTs as a therapeutic approach for hematologic diseases by examining the development of efficient and safe drugs targeting TNTs.

## 1. Introduction

### 1.1. General Considerations on Tunneling Nanotubes

**Figure 1:**
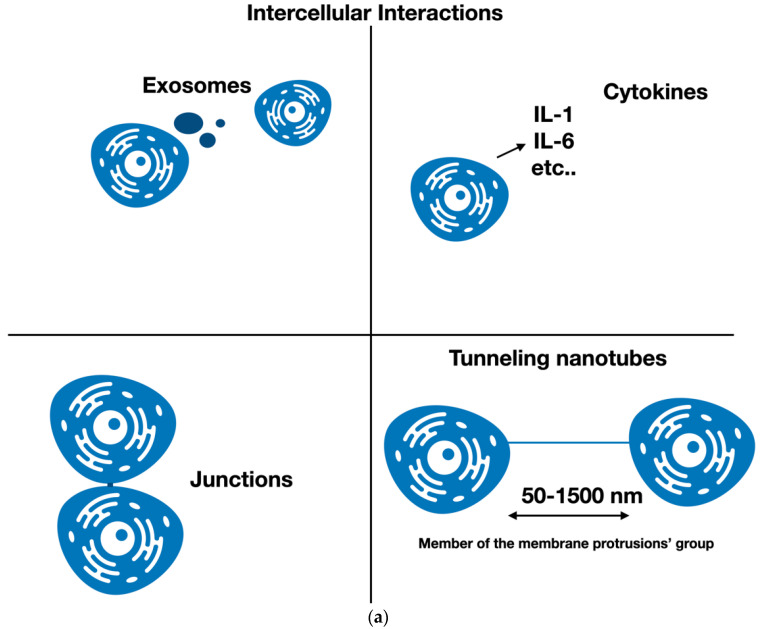

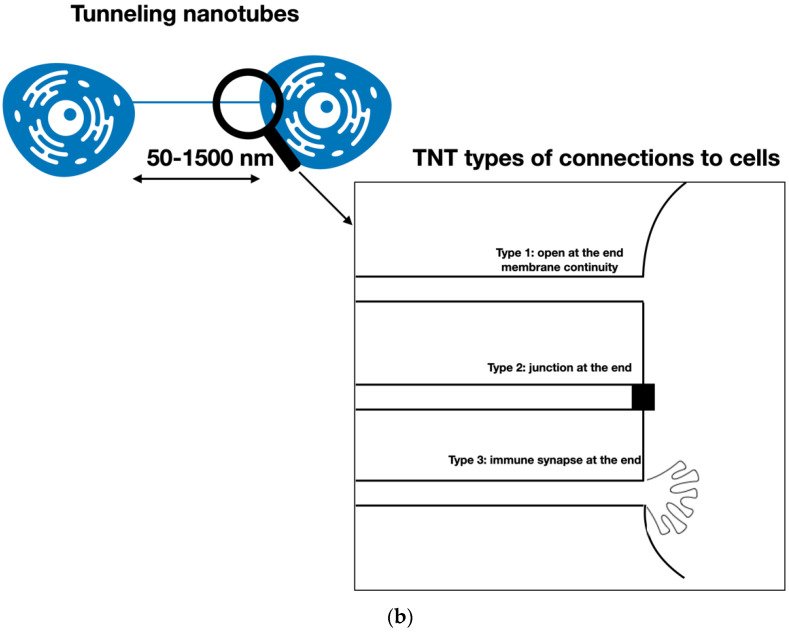
(**a**) Different types of intercellular interaction. Cells can communicate among each other via the release of exosomes and cytokines, by connecting through junctions and, as described in this paper, via tunneling nanotubes (TNTs). TNTs belong to the membrane protrusions group. (**b**) TNT connections to cells could be of 3 types. Some TNTs are open ended at both ends, and so display membrane continuity, while other TNTs are close ended, containing an immune synapse or a junction as gating system.

Intercellular interactions perform an essential action in tissue homeostasis, and are a critical element for cells and tissues growth. In fact, as biological complexity increases, cells must elaborate diverse and more advanced systems to transfer temporal and spatial data. This cell–cell interaction can occur both through the extracellular space, with the production and release of exosomes, cytokines, and other mediators, and through cell–cell contacts that facilitate the association between two cytoplasm (Figure 1a). These types of intercellular interactions are most efficient over brief distances [1,2,3].

Tunneling nanotubes (TNTs) are a new form of direct contact interaction among cells, as they help cells to “talk” directly with each other over lengthier spaces [4,5,6] (Figure 1a). This breakthrough contested the classical view of cells, the basic unit of organisms, as separate elements. The detection of TNTs launched the notion of super-cellularity, which permits the quick stability of metabolic requirements, as well as stress elements and organelles, via long-distance transfers between cells not closely in contact; therefore, TNTs make quick reactions between cells possible despite the diversity of type of cells and tissues [7].

TNTs are cytoplasmic channels, which are members of the group of membrane protrusions, such as filopodia, intercellular bridges, and cytonemes. They regulate the contacts between cells and are independent of soluble factors [8,9]. These elements permit the transfer of cellular content between non-adjacent cells [10,11,12]. TNTs have a length oscillating from 50 nm to 1500 nm [9] and, given a particularly high length/diameter ratio, a TNT is like a one-lane road. Different cell types employ TNTs for specific functions, such as forming a network of cells, causing an intensification of the signal cascade, or to generate a complex communication system that accelerates the transport of substances. [13]. Some TNTs are open at both ends, and so have membrane continuity [14], while other TNTs are close ended, containing an immune synapse or a junction as a gating system [15,16] (Figure 1b).

The development of TNTs involves the active transformation of actin filaments from globular actin (G-actin) into double-helical filament actin (F-actin). The main mediator of the mechanism of actin polymerization is Arp2/3, a protein complex that is stimulated by elements of the Wiskott–Aldrich syndrome protein family (e.g., WASP, WAVE), and is essential for actin nucleation [17].

In addition to F-actin, cytokeratin strings and microtubules are also identified in TNTs in some types of cells [18,19,20].

**Figure 2:**
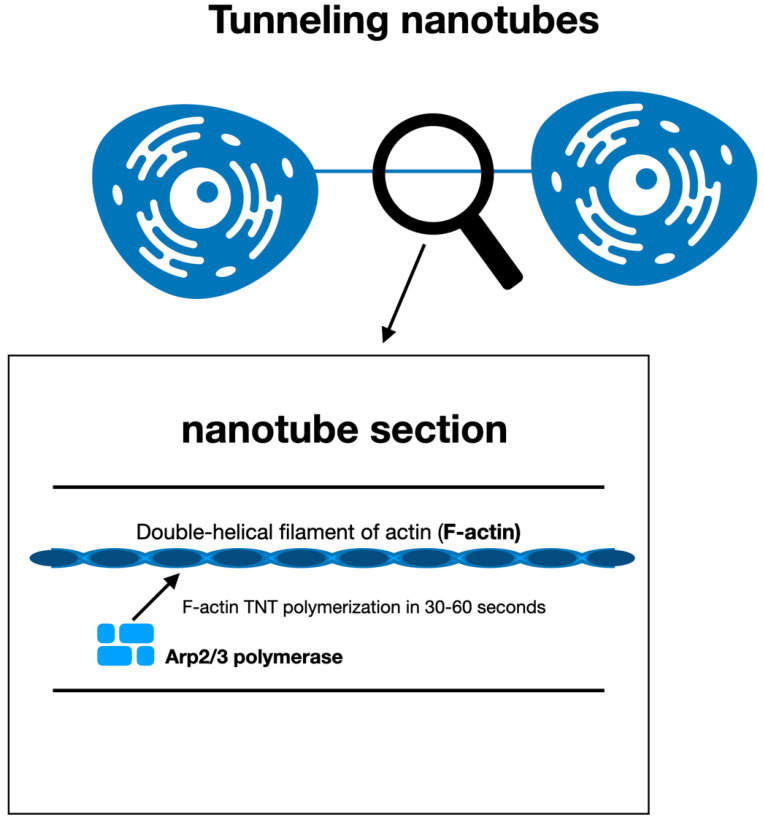
A magnified nanotube section showing the double-helical filament actin (F-actin). The main mediator of the procedure of actin polymerization is Arp2/3, a protein complex. TNTs are able to polymerize and depolymerize quickly in 30–60 s.

F-actin depolymerization substances stop TNT generation, and TNTs are unstable transient formations that can polymerize and depolymerize rapidly in 30–60 s [21,22], with a lifetime oscillating from only some minutes up to numerous hours [23,24,25] (Figure 2).

Different modalities of TNT generation were recognized [25]; the first is the de novo production of TNTs from filopodia-like projections by an actin-directed phenomenon which happens in several minutes [26]. The second is TNT generation during cell–cell disconnection after a direct contact. This system has been reported in immune cells, such as natural killer (NK) cells, macrophages, and T cells, and in rat kidney cells [27]. During the contact, cells generate an immune synapse or fuse, while, with the detachment, a TNT is produced. As mechanical strengths are essential to generating the TNT after membrane contact, an appropriate membrane interaction is indispensable for the generation of TNTs. For instance, T cells generate TNTs only after four minutes of uninterrupted contact [13]. For the duration of this contact, adhesion proteins make available an adequate connecting force for retaining the connection at the ends of TNTs [28]. Furthermore, the extension of TNTs necessitates overcoming the resistance of the actomyosin cortex on the inner surface of the cell membrane, as well as the membrane stiffness.

TNTs operate as intercellular channels for the transfer of elements of diverse dimensions, comprising ions, mitochondria, microvesicles, exosomes, lysosomes, proteins, and microRNAs [13], and via this transport, TNTs are implicated in different physiological and pathological phenomena, such as the immune response, cell proliferation and differentiation, embryogenesis, programmed cell death, pathogen transport, and angiogenesis [29].

It has been also demonstrated that TNTs have an essential effect in the transduction of signals. For instance, Ca^2+^ signals can be transmitted via TNTs between distant cells. In 2010, Wang et al. proved that TNTs mediate depolarization coupling in non-neuronal cells, suggesting that TNTs assist electrical signal transmission [30]. Generally, electrical signals are transported via gap junction or neuronal synapses, which first require a strict contact for cell communication. Conversely, TNTs may transfer these types of signals over long distances [31]. The rapidity of TNT-dependent transfer is due to the forms of transport. For active transfer, the speed oscillates from 0.1 to 8 m/s [32], which is quicker than passive transport along the membrane tube. Besides, the transduction of electrical signals through TNTs occurs in milliseconds [33,34].

Different factors seem able to induce the generation of TNTs, principally, stress situations, such as infections or inflammations, hypoxia, temperature, x-radiation, and UV and hydrogen peroxide exposure [35,36,37,38,39,40,41,42,43], which modify cell viability by damaging the DNA or altering the function of cellular structures. An experiment reported the generation of TNTs after an increase in the number of the reactive oxygen species (ROS) in cells. These stressed cells transmit requests for help to the nearby cells. Therefore, TNTs will be generated by non-stressed cells to form a communication channel. After the transfer of elements such as mitochondria, it is possible to avoid cell apoptosis. From this perspective, TNTs might be considered a tool to increase cell survival during stress [44].

However, other factors seem to contribute to TNT generation. For instance, the strict control of Rho GTPase activity by Guanine nucleotide exchange factors and GTPase-activating proteins indicates that these Rho controllers may participate in TNT generation and clearance [45]. Furthermore, several other pathways influence their synthesis, such as p53-activated pathways, which were stated to be crucial for the generation of TNTs. Cells generate TNT by stimulating p53, which successively increasing epidermal growth factor (EGF) expression. EGF stimulates the Akt/PI3k/mTOR system to provoke the actin polymerization required for TNT synthesis. This procedure is due to the effect of M-sec, a protein able to modify the cytoskeleton. In fact, the increase in M-sec provokes the generation of TNTs, while knock-out M-sec models presented a decrease in TNT generation by as much as one third [46,47]. However, a study demonstrated that TNTs also form independently of p53, and the effect of H2O2 on TNT formation is cell-type dependent and p53 independent [48].

### 1.2. TNTs and Immune System

A separate discussion needs to be had regarding the formation, structure, and function of TNTs in the cells of the immune system. In fact, a different cause of TNT generation might be the Fas ligand receptor in the immune system cells [49]. TNTs may regulate novel controlling systems for immune cell activities, such as chemokine generation, T cell stimulation, antibody production, or phagocytosis.

Spontaneously, B cells develop a large TNT net that includes not only F-actin, but also microtubules. B cell receptor- and lipopolysaccharide-originated stimulation signals block or increase the generation of TNTs. The generation of TNTs may be also controlled by the amount of RAFT gangliosides, which differ in type and amount in immature and mature B cells [50].

Matula et al. reported that adipose tissue-derived mesenchymal stem cells (AD-MSCs) and T cells can transfer cytoplasmic elements via TNTs to induce their immunomodulatory actions [51]. Moreover, the configuration of TNTs is diverse in the different effectors of the immune system. T cell TNTs contain actin, and no microtubules are identified in T cells, which is different with respect to the TNTs detected in macrophages. Furthermore, the TNTs developed between T cells are impermeable to calcium ions, and their configuration is different from the open structures recognized in other cells. T cells can also receive molecules from antigen-presenting cells (APCs) via TNTs to acquire specific antigen-presenting abilities [52]. Different antigens can be transported through TNTs, and this transfer in DCs can be stimulated by CD40L+ CD4+ T cells, promoting the onset of specific T cell responses.

As mentioned above, the TNTs present on the different immune effectors are extremely heterogeneous from a functional point of view. For instance, the TNTs identified in NK cells were reported to cooperate with cells over long distances, causing the lysis of remote target cells. It was also demonstrated that cutting off TNTs can diminish the lysis of target cells [16]. Finally, several cytokines, such as IL-2, IL-12, IL-15, and IL-18, are capable of generating TNTs in NK cells [16].

Thus, TNTs transmit signals that participate in the activation of effectual immune responses [53]. Moreover, TNTs are the perfect tool for the intracellular transport of antigen and MHC–antigen complexes (pMHC) between distant immune cells, enhancing T cell stimulation [54]. It has been demonstrated that MHC class I molecules are transported from one cell to another through TNTs [55]. These findings proved that the TNTs regulated by the HLA-class III-encoded protein LST1 in APCs consent to antigen transport among the immune cells, which would participate in the antigen cross-presentation [56].

Finally, TNTs allow the transport of pro-phagocytic factors, and this might also be relevant for macrophage activity. T cells transport FAS ligands via TNTs, provoking programmed cell death, after which TNTs can be employed to engage macrophages to stimulate self-clearance [57].

In consideration of all that has been reported, a rising number of studies have been performed to exploit the features of TNTs to modify the immune response. For instance, Rainy et al. demonstrated that H-Ras-enriched plasma membrane patches can be transported from B to T cells by stimulating the generation of TNTs, thus augmenting the function of T cells [58].

## 2. TNTs and Cancer

The interaction between tumor cells and neoplastic microenvironments is essential for tumor progression. Tumor cells may stimulate stromal cells to intensify pathways that promote cancer cell proliferation, thus facilitating tumor progression. Polak et al. identified a signal sent from cancer cells to stromal cells via TNTs with the subsequent production of survival-stimulating factors, such as cytokines [59]. Therefore, TNTs might be implicated in an oncogenic microenvironment framework, and have been identified in numerous tumor cells, including breast, ovarian bladder cancer, glioblastoma, and neuroblastoma cell lines [60,61,62,63]. Furthermore, Ady et al. reported that malignant mesothelioma cells display 20-fold to 80-fold more TNTs with respect to normal mesothelial cells [64]. Finally, TNTs are not only recognized in in vitro tumor cell cultures, but also in in vivo cell cultures [65,66].

In vitro experiments employing cancer cell lines have clarified several systems through which TNTs regulate the proliferation and metastasis of tumor cells. TNTs enable homocellular transfer between malignant and normal cells [40,67]. This material could operate through various mechanisms, such as the transfer of substances capable of altering the apoptotic dynamics (pro-growth signals), modifying the metabolism and energy balance, inducing changes in immunosurveillance, or in the response to chemotherapy. For example, TNT has recently been reported to propagate the oncogenic information of the K-RAS mutation among colon cancer cells, favoring the spread of the invasive phenotype in recipient cells. [68]. Moreover, numerous experimentations state that TNTs also permit cancer cells to reset the normal adjacent cells to render them more favorable to the generation of a tumor niche.

Other materials transferred by TNTs could have an effect in neoplastic disease, such as microRNAs (miRNAs) and elements able of altering apoptotic dynamics. miRNAs are small, non-coding, single-stranded RNAs, which have been involved in diverse physiologic and pathologic phenomena, such as the immune response, oxidative stress response, angiogenesis, neural development, DNA repair, and tumor development [69]. It has been described in ovarian tumors that TNTs ease the transfer of oncogenic miRNAs between cancer cells and between cancer and nearby stromal cells [63].

Apoptotic and anti-apoptotic inputs can be also transported via TNTs [16]. The participation of TNTs in apoptotic dynamics was demonstrated in T cells, in which apoptotic signals are transmitted through TNTs, and where immune stimulation induces increased TNT generation. In fact, it was demonstrated that phagocytosis signals are transported from apoptotic to normal cells to aid the immune effectors to identify an injured cellular area [57]. An altered number of the functions of TNTs could consequently change the cellular self-destruction aptitude of the tumoral cells.

Furthermore, angiogenesis is a characteristic of tumors [70,71]. It was evidenced that TNTs may stimulate angiogenesis by creating contacts between pericytes and between pericytes and endothelial cells. For instance, it was reported that pericytes generate TNTs and promote the proliferation and ramification of vessels in glioblastoma tissues. Longer TNTs can link far-away vessels, while short TNTs may join adjacent sprouting vessels. TNTs that link endothelial cells also include lipid drops, which are augmented after VEGF treatment [72,73]. Moreover, miRNA transport between endothelial cells and tumor cells via TNTs has lately been correlated to cancer progression. It was also reported that metastatic cancer cells generated TNT-like bridges with endothelial cells [74].

Finally, a crucial role of TNTs in tumors correlates to bioenergetics. The greater part of human cells employ mitochondria as the principal font of energy. Numerous studies suggest that some cancers, such as endometrial tumors or pancreatic cancer, depend on oxidative phosphorylation (OXPHOS) and augmented mitochondrial metabolism [75,76].

Tumor cells can discharge complete mitochondria or their elements, such as mitochondrial DNA, to the extracellular area [77]. These elements function as damage-associated molecular patterns (DAMPs) that stimulate the immune effectors [78,79], inducing inflammation and immunosuppression, thus modifying the proliferation ability of the cancer [80,81]. Therefore, the modulation of cancer mitochondria is an essential system that helps tumor cells to avoid immunosurveillance [82,83]. Several findings proved that TNTs are the principal transfer mechanism for mitochondria in both normal and cancer cells [84].

Molecular motors are essential for the effective transfer of mitochondria through TNTs [37]. Precisely, myosin Va and myosin X have been identified alongside mitochondria in TNTs [85,86], while certain donor cells show a great expression of the small GTPase Miro1 contained on the external surface of mitochondria [87]. When Miro1 was reduced in MSCs cultured with LA-4 epithelial cells, mitochondrial transport was ineffectual, while Miro1 increase led to the improved capacity of MSCs to transfer mitochondria [88]. Miro1 appears to organize mitochondrial passage along TNTs by supporting the formation of a molecular motor apparatus [89].

The transport of mitochondria via TNTs was also reported between the cancer milieu and tumor cells and between cancer cells and the metabolic actions of the accepted mitochondria, which was displayed in several experiments. TNT-mediated mitochondrial transfer changed the energetic metabolism of the accepting cell, causing increased OXPHOS and ATP generation [90,91]. The achievement of mitochondria by tumor cells provoked an increase in the growth and capacity of progression.

Moreover, resistance to tumor treatment is still a critical problem, causing relapse and decreased overall survival. Several mechanisms that promote chemoresistance have been reported [92,93], and it is recognized that aggressive aptitudes and chemoresistance are correlated to the augmented communication function in tumor cells, and TNTs could have an effect in the onset of chemoresistance. Mitochondrial transfer may induce chemoresistance through different systems, comprising the inhibition of programmed cell death, the decreased production of ROS, and the increased salvaging of cells with altered mitochondria [94]. For instance, mitochondria have been shown to be transported via TNTs between RT4 (less aggressive) and T24 (extremely aggressive) bladder cancer cells with the stimulation of mTOR and other pathways to augment the aggressive potential of RT4 cells [95]. An analogous system of chemoresistance has been reported in tumor cells that were isolated from patients and then treated with doxorubicin [62]. Doxorubicin causes TNT generation in a dosage-dependent manner. Tumor cells that are sensitive to chemotherapy could transfer doxorubicin to resistant cells through TNTs [62]. These findings led to the insertion of antineoplastic drugs to the number of cellular stresses capable of stimulating TNT generation. Moreover, the transport of chemotherapeutic substances not only decreases the amount of drug that the target cells are receiving, but also endangers adjacent normal cells to the damaging actions of doxorubicin, while TNTs increase the rate of survival of cancer cells by reducing the amount of drug in the cellular microenvironment [62]. However, the preferential killing of sensitive cancer cells, decreasing the amount of cancer cells contending for nutrients, improves the ability of resistant cells to proliferate [62].

Lastly, TNTs might mediate other effects, such as the so-called bystander effects [96,97], where non-irradiated cells display a radiation response with augmented programmed cell death and increased DNA damage [98]. It is likely that these effects are due to the TNTs transporting factors which are discharged by irradiated cells to their neighboring cells [99,100].

## 3. TNTs and Hematological Malignancies

The effects of TNT transfer in the tumor microenvironment are critical for the onset, progression, and drug resistance also in hematological tumors [101], as TNTs have an essential role in the interaction between tumor cells and bone marrow-derived cells [21].

**Figure 3:**
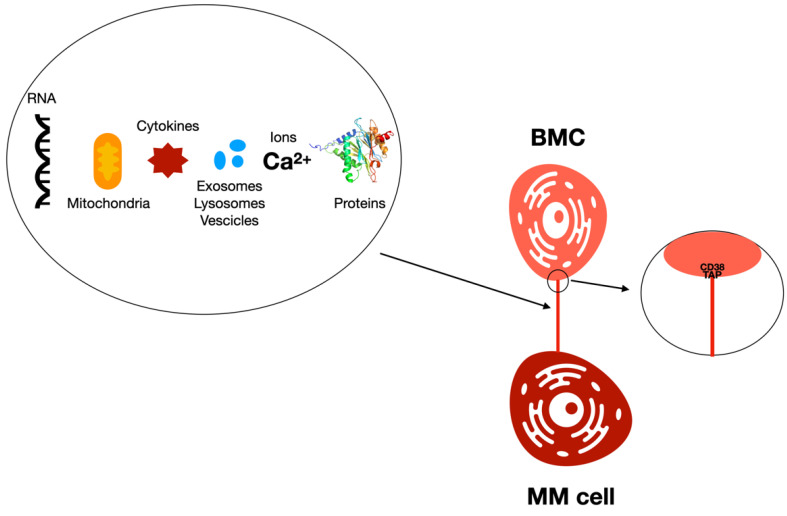
The effects of TNT transfer in the tumor microenvironment are critical for cancer onset and progression. Metabolic changes are indispensable for the beginning and proliferation of MM cells. RNA, mitochondria, cytokines, exosomes, lysosomes, vesicles, ions, and proteins move from one cell to another via TNTs. TNT anchor points (TAP) are portions of membranes from the MM cells that are discovered on the BMSCs after the TNT link is missed. It was stated that CD38 existent on MM cells is essential for TNT generation, as CD38 was present within TAPs on the BMSCs.

An example of a hematological disease in which TNTs appear to play a role is multiple myeloma (MM), the second most common hematologic tumor. It remains incurable for most patients, even though essential progress has been made in the therapy of this tumor [102,103,104,105,106],

Metabolic changes are indispensable for the generation and proliferation of MM cells, and the MM BM milieu sustains MM growth by stimulating mitochondria-derived oxidative phosphorylation (Figure 3). Marlein et al. identified mitochondria within the TNTs connecting BMSCs and myeloma cells [107]. TNTs were calculated by analyzing the amount of TNT anchor points (TAP) that were existent on the membrane of BMSCs. TAPs are portions of MM cell membranes that are discovered on the BMSCs after the TNT link is interrupted. Moreover, it was stated that the CD38 existent on MM cells is essential for TNT generation, as CD38 was present within the TAPs on the BMSCs (Figure 3). Moreover, CD38 is greatly present on MM cells, and it is the target of treatments for MM. The amount of CD38 was recognized to be associated with TNT generation, as an increase in CD38 facilitates mitochondrial transport from BM stromal cells (BMSCs) to primary MM cells [107]. In an in vivo animal study, it has been demonstrated that changing CD38 expression through the shRNA-mediated knockdown of CD38 modifies mitochondrial transfer; moreover, blocking CD38 inhibited mitochondrial transport, caused a reduction in the amount of MM, and enhanced animal survival.

Finally, the transfers mediated by TNTs could also be critical in the onset of organ alterations by MM, such as the occurrence of lytic lesions. In fact, fast intercellular transfer via TNTs has been reported during osteoclastogenesis, and TNTs are identified in osteoclast precursors. The inhibition of TNT generation by blocking actin assembly or with M-Sec RNAi considerably reduced osteoclastogenesis, which was associated with the stimulation of the M-Sec gene, whose expression was enhanced by RANKL administration on an osteoclast precursor cell line [108]. Interfering with this system could be an effective approach for the prevention or treatment of bone disease in patients with MM.

However, most of the studies on the effects of TNTs in hematological diseases concern acute and chronic myeloid and lymphoid leukemias.

Acute myeloid leukemia (AML) is distinguished by the infiltration of the BM by the clonal and inadequately differentiated cells of the hematopoietic system [109].

The destiny of hemopoietic stem cells (HSC) is strongly regulated by cell-intrinsic elements, such as transcriptional and epigenetic controllers, and by cell-extrinsic elements, such as cytokines, ligands, and adhesion molecules [110]. Numerous data have demonstrated the existence of cell-to-cell communications between HSC and the niche cells, such as stromal and endothelial cells, or osteoblasts, which are fundamental for HSC protection and differentiation [111,112].

Moreover, the bone marrow microenvironment (BMME) defends not only HSCs but also their cancerous equivalents, the leukemia-initiating cells (LICs). Inside the communication that happens between HSCs, LICs, and the BMME, the transport of cellular components and of mitochondria is the most relevant to intercellular communication.

The existence of leukemic TNTs has been described, and AML cells have been reported to generate homotypic and heterotypic TNTs with BM cells [113,114]. The latter can transport mitochondria to AML cells, favoring their survival. Generally, as reported above, tumor cells depend on aerobic glycolysis to produce adenosine triphosphate (ATP), as theorized by Warburg in 1956 [115,116], and this is an effect of the stimulation of the oncogenes that activate glycolysis [117]. However, the metabolic activity of AML blasts differs from most other cancer cells in that AML cells are primarily dependent on mitochondrial oxidative phosphorylation for their survival. [118]. It is also evidenced that AML cells have greater mitochondria amounts with respect to normal hematopoietic stem cells [119,120].

Mitochondrial transport was reported in numerous forms of hematological malignancies, where it has a pro-neoplastic meaning [121]. Omsland et al. reported that the TNTs connecting AML cells and stromal cells ease the transport of mitochondria, and AML cells separated from BM produced more TNTs than cells without stromal elements [113]. They discovered that the NF-κB pathway was involved in TNT production and control, studying the occurrence of TNTs on AML cell lines and primary AML cells. Moreover, cytarabine, both alone and with daunorubicin, reduces TNT formation and blocks NF-κB activity, offering further proof in favor of the participation of the NF-κB system in TNT generation. Remarkably, daunorubicin was reported to concentrate into lysosomes in TNTs joining AML cells, supporting the action of TNTs as drug transferring tools [110]. Leukemia-stroma TNTs were reported also to control cytokine secretion, and this might participate in the onset of leukemia [122].

With regards to the systems that control the transit of mitochondria, an essential action appears to be performed by oxidative stress, which has also been reported to support tumor growth and progression [123,124,125]. In AML cells, NOX2 produces superoxide, which pushes bone marrow stromal cells (BMSC) toward AML blasts in the transport of mitochondria via AML-originated TNTs. Furthermore, blocking NOX2 results in the inhibition of mitochondrial transport, and an increase in AML programmed cell death. Adding cytochalasin B to the culture, mitochondria proceed from BMSC to AML blasts essentially via TNTs. Nevertheless, although mitochondrial transport from BMSC to normal CD34+ cells occurs in reaction to oxidative stress, the NOX2 block has no effect on normal CD34+ cell survival. It was suggested that treatment further augments the oxidative stress milieu of the BM caused by the AML blast, and so increases mitochondrial transport. This could be important from the perspective of obtaining minimal residual disease after treatment. It was observed that the few remaining AML blasts may present an exceptionally increased number of mitochondria. This condition would support blast survival and may provoke a relapse [84]. Thus, affecting this specific system may be useful in upcoming approaches designed to decrease the number of relapses from minimal residual disease.

Saito et al. evaluated how the BM milieu modifies the response to OXPHOS reduction in AML by employing a complex I OXPHOS inhibitor, IACS-010759 [126]. Direct communications with BM stromal cells stimulated mitochondrial respiration and increased the transport of mitochondria that originated from MSCs to AML cells through TNTs. Moreover, the reduction of OXPHOS also provoked mitochondrial fission and increased mitophagy in AML cells. As mitochondrial fission is recognized to increase cell migration [127], the authors employed electron microscopy to study mitochondrial transfer from cells to MSCs, and stated that cytarabine augmented the mitochondrial transport stimulated by OXPHOS inhibition. Thus, affecting mitochondrial respiratory activity is a new possibility to proficiently destroy leukemic cells [120,128].

In was also reported that the portion of LICs that have acquired mitochondria during cytarabine administration are more resilient to cell death and possess a greater proliferation capacity [114]. Thus, pharmacological methods modifying leukemic metabolism to an inferior OXPHOS significantly enhance the therapeutic effects of cytarabine [129].

### 3.1. TNTs and Acute Lymphoblastic Leukemia (ALL)

B ALL cells are located in the BM and are able to alter normal hematopoietic stem cell niches [130].

Several studies have recognized TNT generation as a new controller of communication between B cell precursor (BCP)-ALL cells and their BM niche, which facilitates the signaling from ALL cells to MSCs, and modifies the production of chemokines in the BM milieu. A study demonstrated that ALL cells utilize TNTs to transfer communications to MSCs [59]. This event causes the production of pro-survival cytokines, such as interleukin 8, interferon-gamma-inducible protein 10/CXC chemokine ligand 10, and monocyte chemotactic protein-1/CC chemokine ligand 2 [59]. These data suggest that TNTs are essential for the survival of ALL cells, while the interference of TNTs remarkably reduces the leukemogenic processes [130].

Burt et al. studied the MSC niche in adult ALL patients [131]. Primary MSC and MSC cell lines became activated after the administration of cytarabine (AraC) and daunorubicin (DNR), drugs that are able to induce ROS formation. This activation was reduced by administering the antioxidant N-acetyl cysteine. Chemotherapy-stimulated MSC cell lines were studied in a co-culture experimental model with ALL targets. Stimulated MSC blocked treatment-provoked programmed cell death in ALL targets, with mitochondrial transport occurring via TNTs. The decrease in mitochondrial transport through mitochondrial diminution or by interfering with TNT generation and employing microtubule inhibitors, such as vincristine (VCR), avoided the antiapoptotic activity of the stimulated MSC. Steroids also stopped the stimulation of MSC. It was also reported that AraC-induced the stimulation of MSC, mitochondrial transport, and mitochondrial amount increased in an animal model of ALL [131]. These findings suggest a therapeutic strategy for successful therapy with ALL.

**Figure 4:**
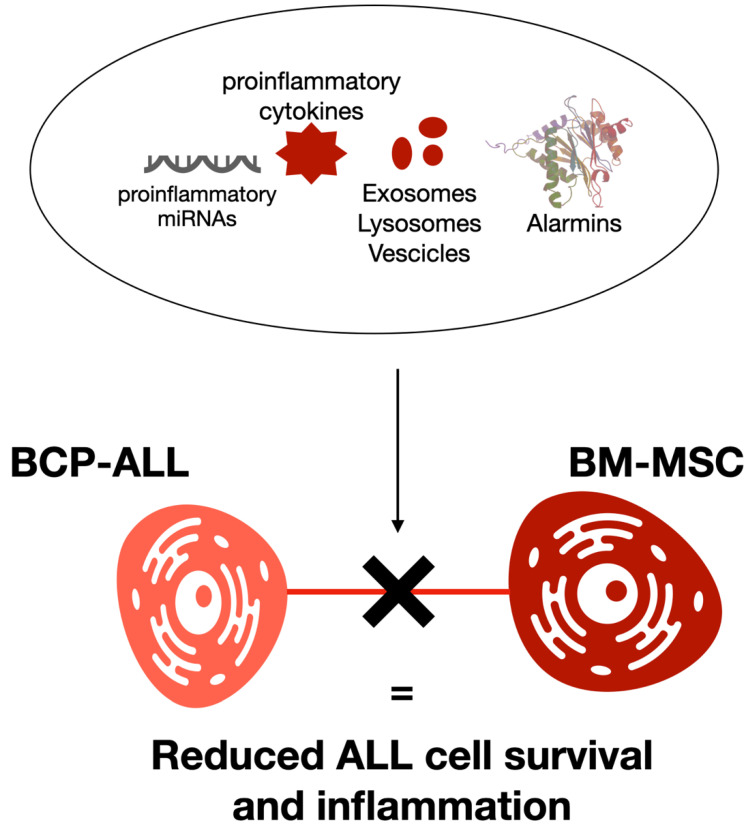
BCP-ALL cells employ TNTs to provoke an inflammatory microenvironmental condition within BM cells. The transfer of pro-inflammatory substances, such as specific microRNAs, alarmins, and cytokines, was involved in tumor development and progression in several tumors. Several cytokines were demonstrated to be involved in leukemia persistence. TNTs were blockaded, reducing this inflammatory condition and reducing ALL cell survival.

A recent study by Manshouri et al. demonstrated the generation of chemoresistance against Janus kinase 2 (JAK2) inhibitors by BM-MSC-produced chemokines in JAK2-mutated cells [132]. BCP-ALL cells employ TNTs to provoke an inflammatory cytokine environment within the BM milieu [133]. Several of these cytokines are involved in leukemia persistence [134]. This inflammatory condition was partially overturned, and ALL cell survival was reduced when TNTs were blocked [135] (Figure 4). Furthermore, it was reported that TNT signaling from MSCs to ALL cells might also provoke the chemoresistance of leukemic cells (Figure 4). For example, TNTs have been reported to transfer drug efflux pumps, such as P-glycoproteins, toward tumor cells [92].

A unique sort of transfer of mitochondria due to TNTs is that correlated to autophagy, a condition that decomposes and reutilizes harmed cellular elements, such as proteins and organelles. This procedure has been involved in tumor onset and inhibition. Autophagy is operated by vesicles called autophagosomes, which are formed by an actin cytoskeleton, and TNT generation is also due to actin. Remarkably, autophagy stimulation via the starvation of cells has been recognized to increase the generation of TNTs, proposing a connection between these two events. Moreover, TNT transfer causes the production of pro-survival cytokines, and autophagy is also a controller of cytokine production, and this signaling is regulated by mitochondrial ROS and DNA [136,137,138]. The transport of autophagosomes and mitochondria from leukemic cells to MSCs might consequently justify the liberation of helpful elements by the tumor milieu. Remarkably, the production of the adhesion molecule ICAM1 is recognized to be increased by cytokines and ROS [139], and its transfer to MSCs may facilitate TNT action. A study demonstrated that autophagosomes are transferred via TNTs, and the transport of autophagosomes and mitochondria from ALL cells to MSCs might be an essential system that leukemic cells employ to modify the BM milieu. This study confirms that leukemic cells transport autophagosomes to MSCs to increase autophagy-provoked cytokine production, and are able to modify the progression of ALL [140].

### 3.2. TNTs and T Cell Acute Lymphoblastic Leukaemia (T-ALL)

T cell acute lymphoblastic leukemia is an aggressive hematological malignancy. Despite the high cure rate of T-ALL, chemoresistance remains a critical clinical barrier.

T-ALL BM-MSCs defend leukemic cells from treatment, although the causal mechanism is unknown. In a study on Jurkat cells, chemotherapy resulted in intracellular oxidative stress. Jurkat cells transferred large numbers of mitochondria to MSCs, but withdrew a small number of mitochondria from MSCs, thus causing chemoresistance. This mitochondrial transfer procedure was performed via TNT. Furthermore, Jurkat cells adhered to MSCs in the culture medium, and this process was due to ICAM-1. The use of an antibody against ICAM-1 reduced the amount of adhering Jurkat cells, and increased chemotherapy-caused cell death. Inhibiting mitochondria transport with cytochalasin D reduced the ability of MSCs to defend T-ALL cells. Therefore, the reduction of T-ALL cell/MSC adhesion-originated mitochondria transfer may be a new method for T-ALL therapy, and increasing mitochondrial ROS represents a possible means of destroying T-ALL cells [141].

A role of TNTs has been demonstrated in other forms of hematological malignancies involving T lymphocytes. Adult T cell leukemia/lymphoma (ATL) is a malignancy of peripheral T lymphocytes provoked by human T cell leukemia virus type 1 infection (HTLV-1). This leukemia is extremely resistant to treatment, and it is estimated that at least 5–10 million patients are infected with HTLV-1, the first human oncogenic retrovirus detected [142].

In contrast to HIV-1, cell-free HTLV-1 in the blood of HTLV-1 infected patients is poorly infectious [143]. Thus, cell-to-cell communication is the principal modality of HTLV-1 transmission and diffusion [144,145]. Therefore, systems able to decrease cell-to-cell virus transmission have the possibility to diminish the viral burden and the onset of leukemia. Two diverse modalities of cell-to-cell connections have been reported in HTLV-1 diffusion. The virological synapse is a virus caused by strong cell-to-cell connection, generating a synaptic intercellular fissure permitting viral transmission. This provokes a polarization of the microtubule-organizing center in the giver cell to the virological synapse [146]. For longer distances, cellular channels have been described, and the diffusion of HTLV-1 via these forms of cell-to-cell contacts could offer a defense from identification by the immune system [147].

Omsland et al. stated that HTLV-1-bearing cells are connected by TNTs [145]. Moreover, TNTs connect infected and uninfected T cells, and the viral proteins Tax and Gag are present in these TNTs. TNT generation is stimulated by HTLV-1 protein p8. The administration of cytarabine to MT-2 cells decreases the number of TNTs and TNT generation stimulated by the p8 protein. Thus, cytarabine could be a new anti-HTLV-1 treatment able to interfere with viral diffusion [148].

### 3.3. TNTs and Chronic Myeloid Leukemia (CML)

The communications between cells due to TNTs are also relevant in chronic myeloid leukemia (CML), a myeloid stem cell malignancy due to the tyrosine kinase BCR-ABL1 fusion protein originated from the chromosomal translocation *t* (9;22) [149].

Numerous reports demonstrated that stromal and leukemic cells interrelate bidirectionally, to sustain leukemogenesis [150,151,152]. Furthermore, cellular interconnections are essential in the onset in the stroma-originated chemoresistance of CML.

TNTs have been suggested to be involved in CML onset and in the treatment response [153]. Kolba et al. described a direct transport of vesicles through TNTs from stromal to CML cells, providing a defense against imatinib-induced programmed cell death. Several specific proteins with effects in cell survival are transported with these vesicles [154].

A different study evaluated TNTs in CML cells after the administration of tyrosine kinase inhibitors (TKIs) and interferon-α (IFNα) [155]. It was demonstrated that CML cells from patients in chronic phase or from blast crisis phase cell lines, Kcl-22 and K562, generated scarce or no TNTs. The administration of imatinib increased TNT generation in both Kcl-22 and K562 cells, while IFNα or nilotinib worked in Kcl-22 cells only. Ex vivo treated cells from CML subjects demonstrated limited modifications in TNT generation analogously to BM cells from normal subjects. Remarkably, in vivo nilotinib administration in a Kcl-22 experimental animal model caused morphological variations and the occurrence of TNT-like formations in the Kcl-22 cells. These findings reveal that CML cells present small amounts of TNTs, but chemotherapeutic agents increase TNT generation. To confirm the participation of BCR-ABL1 in TNT generation, a doxycycline-inducible BCR-ABL1 Ba/F3 cell system was used. The fact that the administration of imatinib did not cause an increase in TNT formation in BCR-ABL1 cells presenting Ba/F3, but rather in Ba/F3 cells alone, indicates that the increase in TNT most likely involves other elements besides the inhibition of BCR-ABL1. This was also demonstrated by the evaluation of intracellular signaling through mass cytometry, in which known BCR-ABL1 targets, such as phospho-STAT-5, were reduced by TKI administration, regardless of the reported differences in TNT response [155].

The therapeutic effects of TKIs and IFNα in CML may thus be more dependent on leukemia–stroma and cell–cell communications than before estimated.

## 4. Conclusions

The participation of TNTs in various pathological conditions makes them an essential target for treating diseases such as leukemia [156]. Recognizing the molecular bases for TNT generation and the type of molecular information transferred between leukemic and BM cells through TNTs in vivo represents a crucial field for pharmacological experimentations. Our ability to influence TNTs in vivo will be helpful to attain an efficient management of hematologic malignancies.

Numerous substances affecting different pathways, such as NF-KB and mTOR, or blocking actin polymerization, thus causing the reduction of TNTs formation, have been identified, including cytochalasin D, cytarabine, latrunculin A and B, daunorubicin, everolimus, metformin, nocodazole CK-666, ML-141, 6-thio-GTP, BAY-117082, and octanol [153]. The administration of these molecules seems appropriate for antileukemic treatment, reducing the transport of biological substances between leukemic cells and between leukemic and microenvironment cells.

Moreover, TNTs can also offer an efficient instrument for long-range cellular drug delivery [64].

In the context of studies on the use TNTs as tools for drug transport and delivery to leukemic cells, it has been reported that polymeric nanoparticles can be transported through TNTs. This strategy, combining polymeric nanoparticles as a transmitter and TNTs as a transferer for antileukemic treatment, would make the delivery of the drug to all the cellular elements of the BM uniform, especially in a hypoxic context [157].

However, the modification of TNT functions can be used to achieve goals diametrically opposite to those reported above. It could be useful to use the ability of TNTs to support cell viability as part of leukemic treatment: this strategy could be used in hematological patients undergoing stem cell transplantation, whose main constraint is the senescence reached by the transplanted cells, reducing their effectiveness over time [158].

Nowadays, only a limited number of substances, such as arachidonic acid and doxorubicin, have been demonstrated ta to increase TNT generation in tumor cells [70]. Identifying TNT inhibitors seems more feasible than finding drugs capable of stimulating TNT generation. Actin inhibitors have been extensively employed in in vitro experiments. Nevertheless, these substances are difficult to use as drugs due to their cytotoxic properties [159,160,161]. In fact, there can be many possible negative effects with the clinical use of molecules capable of modifying TNT-mediated transfer. In fact, TNTs are principally constituted by actin, which is crucial for different cellular functions and for the preservation of the cytoskeleton: we must target only pathological systems, while protecting normal elements and functions.

Molecules exclusively affecting the proteins that control TNT generation may be those with less toxicity. In some reports, the number and function of TNTs could be decreased by the short hairpin RNA (shRNA)-derived knockdown of CD38 in MM cells [107], by altering the IL-10/STAT3 pathway in macrophages [162], or by inhibiting Ca^2+^/calmodulin-dependent protein kinases II in neuronal cell lines [163]. In any case, it is challenging to identify wide-spectrum inhibitors for the various TNT-generating mechanisms. A different approach is to destroy TNT links by altering the generation or the activity of some cell adhesion proteins, such as cadherin [28]. Moreover, M-sec, a controller of TNT generation, has also been suggested as a possible target to influence mitochondrial transport; TNF-alpha inhibitors, such as those employed for the treatment of autoimmune diseases, may decrease TNT generation, as M-sec is TNF-alpha inducible [164].

Other experiments have demonstrated that metformin or the mTOR inhibitor, everolimus, reduce TNT generation in vitro [36], while cytochalasin B and D block the actin polymerization that inhibits TNT generation [165]. Nevertheless, as reported above, as inhibitors, they are too unspecific, lacking the essential specificity for successful therapeutic applications. Cytarabine was also described to block TNT generation, and it is presently used to treat different forms of leukemia.

In any case, numerous investigations have been launched to evaluate the inhibition of TNT generation as a possible oncological treatment. The initial findings have displayed that the reduction of the TNT-derived transport of the mitochondria causes an increase in chemotherapy-caused cell death and in a more prolonged animal survival [141]. Thus, it seems that inhibiting TNT connections could be a useful approach for cancer treatment.

On the contrary, as mentioned above, we must stimulate the generation of TNT if the contact derived from TNT allows the transfer of molecules capable of repairing damaged healthy cells [166]. The capacity of cells to utilize TNTs as a channel to help distressed cells might be a biological mechanism for cell or tissue self-repairing.

The manipulation of TNTs could also be useful to improve the spread of antineoplastic drugs. In fact, the distribution of macromolecular drugs in tumor tissues generally is slow, and scarcely reaches all target cells [167]. The use of antineoplastic delivery via TNT could be a new method for administering drugs in a highly specific way, and could be an effective modality for the circulation of anticancer substances among related tumor cells [167].

Notwithstanding these encouraging possibilities, other problems for therapeutic use of TNTs remain. One such challenge depends on the instability of these formations, as they are susceptible to several types of stress, such as light, trypsinization, mechanical stress, chemicals, and transfection, all of which can provoke the break of these formations. These problems are evident in in vitro models. Moreover, when attempting to reproduce the in vivo tumor milieu, several signaling components, such as exosomes and microvesicles, can have a mystifying effect on results [153].

To lock the break between our present comprehension and future clinical treatments, we need better knowledge of TNT-dependent communication, and new perspectives for detecting other TNT generation pathways would permit us to target them in treating an increasing number of TNT-involved malignancies [168]. Nonetheless, the recent advances in screening TNT inhibitors may accelerate the advent of a new epoch for TNT research and translational medicine in the treatment of hematologic diseases.
